# Predicting the Potential Distribution of the Endangered *Pyrethrum tatsienense* in China Using an Optimized Maxent Model Under Climate Change Scenarios

**DOI:** 10.1002/ece3.70503

**Published:** 2024-11-03

**Authors:** Duo Ping Zhu, Liu Yang, Yong‐hua Li, Pei Huang, Bin Yao, Zhe Kong, Yangzhou Xiang

**Affiliations:** ^1^ Institute of Ecological Conservation and Restoration Chinese Academy of Forestry Beijing China; ^2^ State Forestry Administration Dunhuang‐ Kumutage Desert Ecosystem Location Research Station Dunhuang China; ^3^ Institute of Desertification Studies Chinese Academy of Forestry Beijing China; ^4^ State Key Laboratory of Tree Genetics and Breeding Institute of Ecolog Conservation and Restoration Chinese Academy of Forestry Beijing China; ^5^ Foreign Environmental Cooperation Center of Ministry of Ecology and Environment of China Beijing China; ^6^ School of Geography and Resources, Guizhou Provincial Key Laboratory of Geographic State Monitoring of Watershed Guizhou Education University Guiyang China

**Keywords:** climate change, different climate conditions, ENMeval, maximum entropy model, *Pyrethrum tatsienense*, suitable habitat distribution

## Abstract

Climate change can significantly impact the ecological suitability and diversity of species. *Pyrethrum tatsienense*, a critically endangered species in China, requires a thorough understanding of its habitat distribution and the environmental factors that affect it in the context of climate change. The Maxent algorithm was used to examine the key factors influencing the distribution of *P. tatsienense* in China, using data from 127 species occurrences and environmental variables from the Last Interglacial (LIG), Last Glacial Maximum (LGM), Mid‐Holocene (MH), current, and future scenarios. The Maxent model was optimized utilizing the R package ENMeval, providing the most accurate predictions for suitable habitats across various scenarios. Results show that suitable regions for *P. tatsienense* encompass approximately 15.02% (14.42 × 10^5^ km^2^) of China, predominantly on the Qinghai‐Tibetan Plateau. The mean UV‐B of the highest month (UVB3: 39.7%), elevation (elev: 28.7%), and the warmest season of precipitation (Bio18: 17.4%) are the major limiting factors for suitable habitat. The optimal species distribution ranges are identified as > 7500 J m^−2^ day^−1^ for UVB3, 2700–5600 m for elev, and 150–480 mm for Bio18. Predictions for the historical climate indicate the presence of refugia at the junction of Sichuan, Tibet, and Qinghai. The MH predictions show an increase in climatic suitability for *P. tatsienense* compared to the LIG and LGM, with an expansion of suitable areas westward. Future climate change scenarios indicate that the potential suitable habitat for *P. tatsienense* is expected to increase with increasing radiative forcing, with higher latitude regions becoming new marginally suitable habitats. However, predicted environmental changes in western Tibet may drive the loss of highly suitable habitats in the future. These findings enhance our understanding of how environmental factors impact the habitat suitability of *P. tatsienense* and provide valuable insights for developing effective management and conservation strategies for this important species.

## Graphical abstract

This study used the ENMeval function‐optimized MaxEnt to predict the potential suitable area for *Pyrethrum tatsienense* under climate change scenarios. The lowest month of Mean UV‐B of the highest month (uvb3), elevation (Elev), and the warmest season of precipitation (Bio18) are three identified major factors limiting the distribution of the *P. tatsienense*. Reconstruct and predict the historical and future geographical distribution pattern changes of *Pyrethrum tatsienense*. Lay a theoretical foundation for the study of flora evolution on the Qinghai‐Tibetan Plateau.

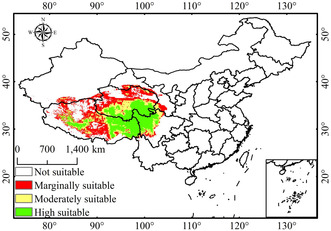

## 1 Introduction

Climate change, driven by anthropogenic activities such as agriculture and industry, has caused significant spatial and temporal environmental changes (Hughes 2000; Lin et al. 2014; Solomon et al. 2007). Projections indicate a global average warming of 1.5°C–5.2°C by 2100 compared to 1850–1880, under different Shared Socioeconomic Pathways (SSP) scenarios (Gidden et al. 2019; Nazarenko et al. 2022). On the ecologically fragile Qinghai‐Tibetan Plateau (Bai et al. 2013; Yao et al. 2000), temperature and precipitation are the primary bioclimatic variables impacting vegetation dynamics (Sun, Cheng, and Li 2013). In addition, topographic features such as elevation and soil factors like soil pH and soil organic carbon are critical for plant growth. Climate change and other environmental factors significantly impact the spatial distribution patterns of species globally, as well as their physiological and ecological characteristics (Bellard et al. 2012; Hoffmann and Sgrò 2011; Walther et al. 2002). Therefore, understanding how vegetation responds to these changing conditions is crucial to developing effective species conservation strategies (Yang et al. 2013).

**FIGURE 1:**
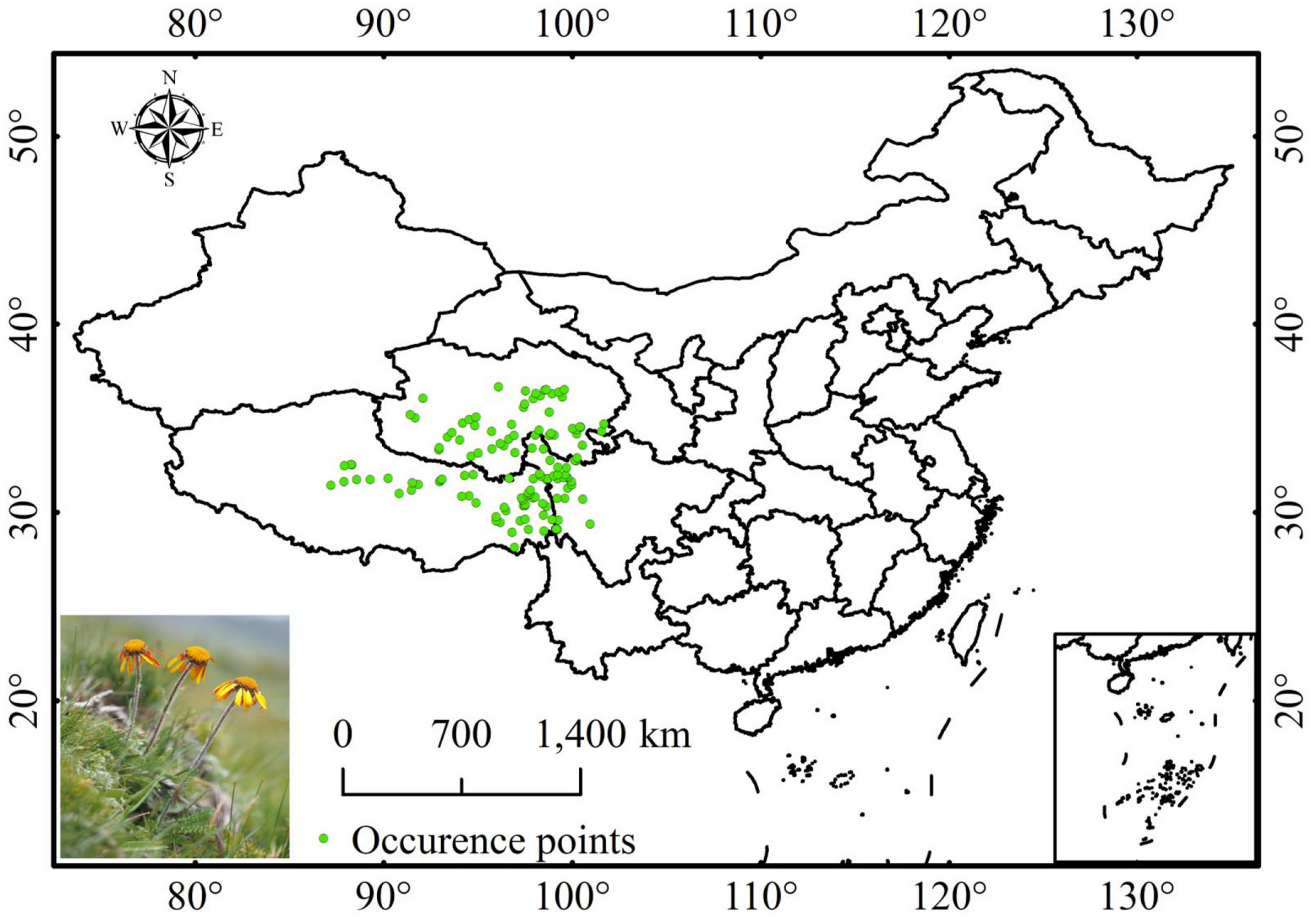
Distribution records of *Pyrethrum tatsienense* in China.

*Pyrethrum tatsienense* is a perennial herb species in China, classified as a critically endangered plant on the Chinese Rare and Endangered Plants Red List (Figure 1). It is primarily found at altitudes of 3500–5200 m on the Qinghai‐Tibetan Plateau, spanning the provinces of Tibet, Qinghai, Yunnan, and Sichuan (Li et al. 2002). This herb grows on slopes, rocky soils, alpine meadows, and shrubs. Known for its medicinal properties, it has heat‐clearing, moisture‐regulating, and anti‐inflammatory effects and has been used to treat hepatitis, headaches, cranial trauma, ulcers, and injuries (Lin et al. 2014; Wei, Zhong, and Sang 2020). Most research on *P. tatsienense* has focused on its pharmacological activities and chemical components, but there is still a lack of knowledge about its phenology and response to climate change.

Many methodological approaches have been used to examine the spatial distribution and habitat suitability of species, as well as their response to environmental predictors (Guisan and Thuiller 2005; Phillips and Dudík 2008). Among these, the Maxent model stands out due to several advantages: it performs well with small samples and incomplete datasets, has short run times, is easy to operate, and yields accurate results (West et al. 2016; Zhang et al. 2018). Consequently, it has been widely employed to predict the potential distribution of various species under historical, current, and future climate scenarios (Hernandez et al. 2006; Ortega‐Huerta and Townsend Peterson 2008; Phillips, Anderson, and Schapire 2006).

In this study, the Maxent model, optimized by the ENMEVAL function, was used to predict the effects of bioclimatic variables on the physiology and ecology of *P. tatsienense* under historical, current, and future climate scenarios. The objectives of the study included: (1) reconstructing the historical geographical distribution patterns of *P. tatsienense*; (2) understanding the impact of environmental changes on its habitats; and (3) predicting future changes in its habitats. The findings aim to provide a reference for the rational layout of *P. tatsienense* production, improvement of planting systems, and development of strategies to cope with climate change.

## 2 Data and Methods

### 2.1 The Existing Records of *Pyrethrum tatsienense*

This study collected existing specimen records of *P. tatsienense* from the Chinese Virtual Herbarium database (https://www.cvh.ac.cn/) and the Global Biodiversity Information Facility (https://www.gbif.org/). By entering the Latin name of *P. tatsienense* in these databases and selecting the region of China, the latitude and longitude of its locations were obtained (accessed on 15 September 2022). The geographical data were carefully analyzed to reduce errors in the *P. tatsienense* records, and specimens with unclear geographic coordinates, classification information, or duplicate coordinates were removed. A 10 × 10 km grid was created in ArcGIS, retaining only one record per cell. The resulting presence‐absence grid contained 127 unique species cell units from a total of 130 records (Figure 1).

### 2.2 Environmental Variables

Climate data for the Last Interglacial (LIG), Last Glacial Maximum (LGM), Mid‐Holocene (MH), current (average from 1970 to 2000), and future projections (averages for 2041–2060 and 2061–2080, corresponding to the 2050s and 2070s) were obtained from the WorldClim database (http://worldclim.org) at a resolution of 2.5 arcminutes. Radiation data, including UVB1‐6 (Manceur et al. 2014), were sourced from the global UV‐B radiation database (gIUV, https://www.ufz.de/gluv/). Soil factors were obtained from the Center for Sustainability and the Global Environment database (http://www.sage.wisc.edu/atlas/index.php), and topographic data (elevation) were also sourced from WorldClim. All datasets were standardized to the same coordinate system and resampled to a resolution of 5 km.

Paleoclimate data for the LIG, LGM, and MH were obtained using the Community Climate System Model version 4 (CCSM4) general climate system model (Gent et al. 2011). CCSM4 is highly effective for predicting the impact of climate change on the distribution of flora and fauna, excelling in precipitation forecasting, particularly for Southwest China (Geng et al. 2022; Yang et al. 2021). The CCSM4 experiments incorporated both anthropogenic and natural forcings for the 20th century, involving evolving greenhouse gases, tropospheric ozone, stratospheric ozone, and sulfates. CCSM4 is widely used by Chinese researchers for climate modeling (Zhao et al. 2021).

Future climate data are based on the sixth phase of the Coupled Model Intercomparison Project (CMIP6), which offers simulations that are closer to actual observations and improve the accuracy of temperature and precipitation forecasts in China. The BCC‐CSM2‐MR model developed by the Beijing Climate Center was selected for this study due to its high accuracy in estimating global climate change (Shi et al. 2023). SSPs provide a framework that better reflects the correlation between socioeconomic development and climate scenarios (Moss et al. 2010). The selected future climate scenarios are SSP126, SSP370, and SSP585. SSP126 (Green Growth Paradigm) predicts a global temperature increase of 3°C–3.5°C by 2100. SSP370 represents a development pathway characterized by strong regional competitiveness, forecasting a temperature increase of 3.9°C–4.6°C by 2100. SSP585, based on SSP5 (fossil‐fuel‐driven development), projects global warming of 4.7°C–5.1°C by 2100 (Fan et al. 2022; Hausfather 2020). Research indicates that considering both the lowest and highest SSP limits provides better predictions of future conditions (Jamal et al. 2021; Xu et al. 2022).

Initially, a total of 28 environmental factors were selected, including bioclimatic, radiation, soil, and topographic factors. The Jackknife method was used to calculate variable importance and quantitatively assess the impact of these environmental factors on the geographic distribution of *P. tatsienense*. Spearman's correlation was used to evaluate the collinearity of environmental factors. Following previous research recommendations, if two variables showed a high correlation (*r* > |0.80|) (Graham 2003; Wei et al. 2024), one was excluded to mitigate collinearity. Considering the biological characteristics of *P. tatsienense*, the most ecologically important environmental factors were retained. Ultimately, 10 environmental factors were kept: 5 bioclimatic variables, 2 radiation variables, 2 soil variables, and 1 topographic variable.

### 2.3 Maxent Model

When simulating species distribution, Maxent typically runs with default parameters (Maxent version 3.4.1), which can lead to overfitting and high complexity, thus reducing the accuracy of the study results (Shi et al. 2024). To address this, the Maxent model in this study was optimized by adjusting the feature combination (FC) and the regularization multiplier (RM) using the R package ENMeval (Phillips, Anderson, and Schapire 2006). The RM was varied between 0.5 and 4 in increments of 0.5, and six types of FC were used: L, LQ, H, LQH, LQHP, and LQHPT. The ENMeval package tested these 48 parameter combinations and evaluated model complexity and fit using the Akaike Information Criterion Correction (AICc). Models with ΔAICc = 0 were considered the best. Additionally, ENMeval evaluated the model's fit to local species distribution points by comparing the mean Area Under Curve (AUC) difference between training and test sets (avg.diff.AUC) and the mean 10% training omission rate (avg.test.or10pct) (Muscarella et al. 2014).

The optimized model algorithm was used to evaluate the suitable habitat of *P. tatsienense* based on the relationship between the species' actual distribution and geographic data (Elith et al. 2011; Zhao et al. 2022). This model algorithm incorporates various feature classes, and the selection of feature types and regularization multiplier significantly affects the accuracy of the Maxent model, especially when simulating habitats for species with small sample sizes. Evaluating the optimal combination of feature classes and regularization multiplier can enhance the prediction of species distribution (Morales, Fernández, and Baca‐González 2017). For this study, 75% of the species data were used for model training, and the remaining 25% were used for model testing (Amiri et al. 2022). The probability of species presence was estimated based on the environmental variables of each grid unit. The maximum number of background points was set to 10,000, and the model ran 500 iterations until convergence (Li, Fan, and He 2020; Xu et al. 2022). Other Maxent model parameters were set according to the Maxent model tutorial.

The optimal model selection was based on threshold‐independent indicators, including model calibration accuracy evaluation and identification accuracy. The area under the receiver operating characteristic curve (AUC) determined the identification accuracy (Elith et al. 2011; Zhao et al. 2022). An AUC value of 0.9 or higher indicates a suitable model (Franklin 2010). The relative importance of environmental variables was examined using the jackknife test (Merow, Smith, and Silander Jr 2013; Phillips, Anderson, and Schapire 2006). The model output value, ranging from 0 to 1, was divided into four categories: not suitable (0–0.2), marginally suitable (0.2–0.4), moderately suitable (0.4–0.6), and highly suitable (0.6–1.0) (Wang et al. 2021).

## 3 Results

### 3.1 Optimal Model and Model Evaluation

**FIGURE 2:**
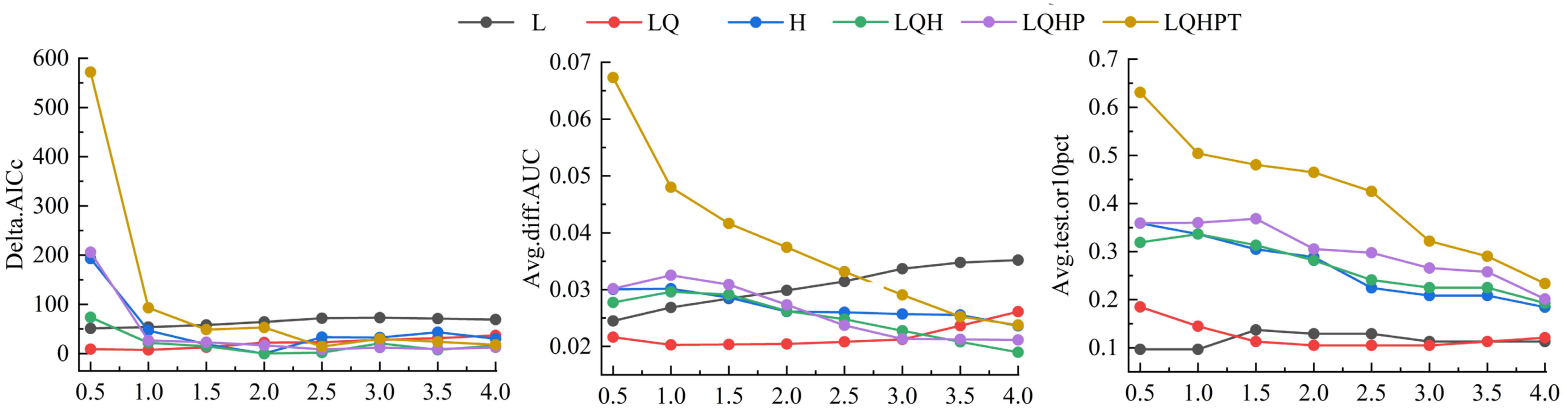
Variations in AICc, avg.diff.AUC, and avg.test.or10pct parameters in the optimized model.

The Maxent model was used to predict the suitability distribution of *P. tatsienense* in China. Under the default parameter settings (RM = 1, FC = LQHPT), the delta AICc was 92.95, avg.diff.AUC was 0.05, and avg.test.or10pct was 0.50. When the RM was adjusted to 2 and FC to H, the delta AICc decreased to 0. In the optimized model, the avg.diff.AUC and avg.test.or10pct values were reduced by 45.56% and 42.75%, respectively, compared to the default model (Figure 2). These optimized parameters significantly enhanced the model fit and transferability. Consequently, RM = 2 and FC = H were selected as the final parameters for this study. In the optimal model, the AUC values for the training and testing datasets were 0.980 and 0.977, respectively, indicating that the Maxent model was highly accurate and reliable in predicting the potential habitat of *P. tatsienense*.

### 3.2 Critical Environmental Variables

**FIGURE 3:**
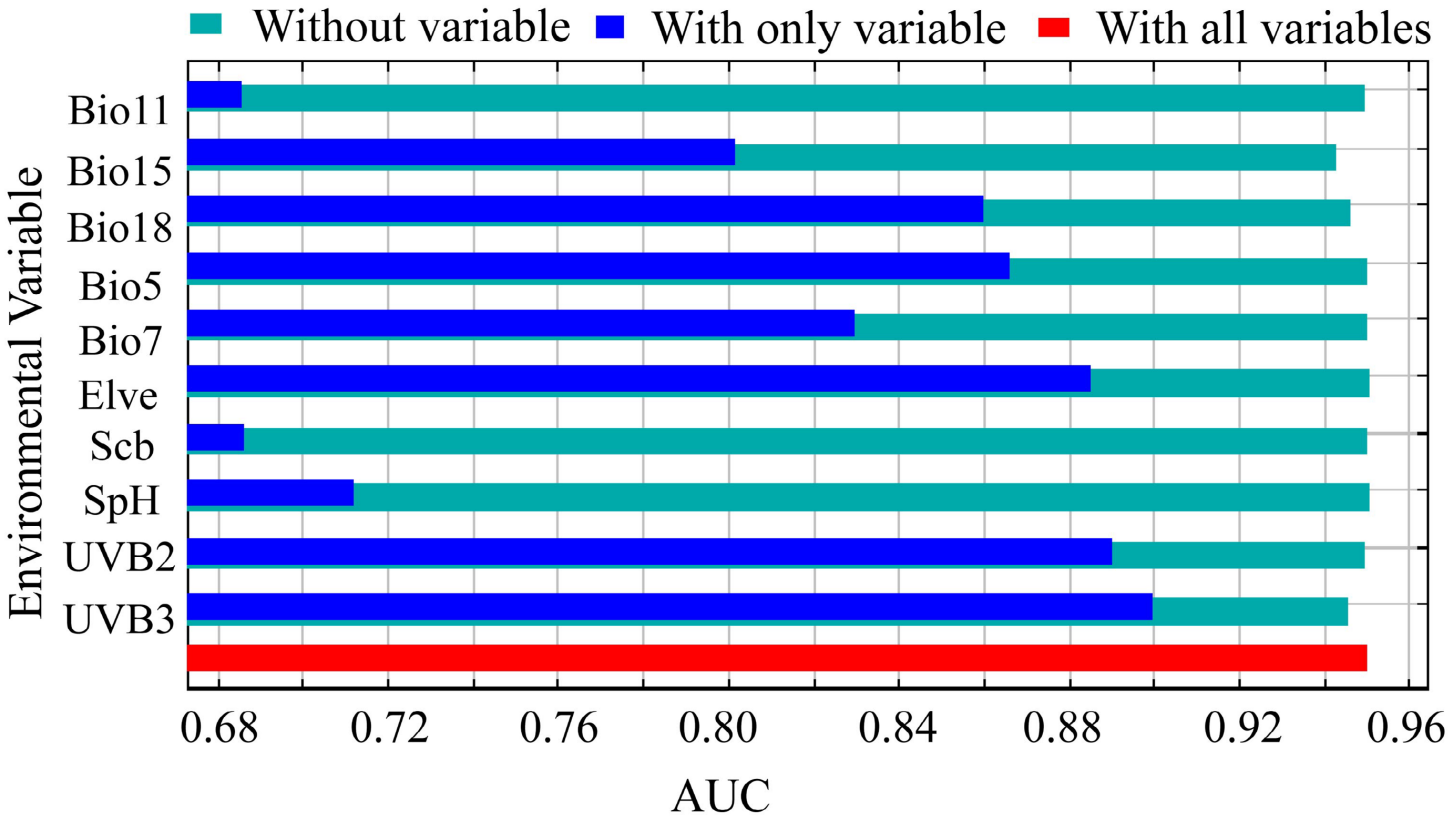
The result of the jackknife test of environmental variables.

**FIGURE 4:**
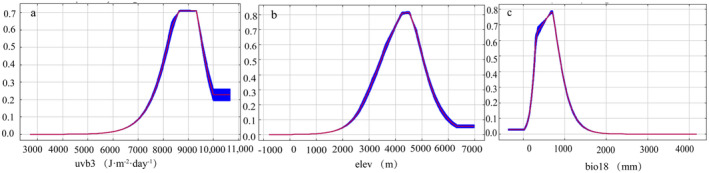
The mean UV‐B of the highest month (UVB3), elevation (elev), and warmest season precipitation (Bio18) range in China. The curve shows the average response (red) and +/− a standard deviation for 10 repeated Maxent runs.

The Maxent model based on variable importance indicates that the month with the mean UV‐B of the highest month (UVB3: 39.7%) was the most important variable, followed by elevation (elev: 28.7%) and warmest season precipitation (Bio18: 17.4%). Together, these three environmental variables contributed 85.8% to the model (Table 1), highlighting their importance in the distribution of *P. tatsienense*. The results of the jackknife test based on individual environmental factors further confirmed the importance of these variables, with the normalized AUC values of UVB3 and UVB2 being the highest (Figure 3), indicating that radiation plays a crucial role in determining the suitable habitat for *P. tatsienense*. Following that are elevation (elev) and precipitation (Bio18, Bio5), as *P. tatsienense* prefers warm and humid environments. Therefore, UVB3, Elve, and Bio18 are the key environmental factors influencing the suitable distribution of *P. tatsienense*. The response curves in Figure 4 were used to determine the thresholds for these key variables (UVB3, elev, and Bio18). The suitable habitat for *P. tatsienense* corresponds to a UVB3 value greater than 7500 J·m^−2^·day^−1^ (Figure 4a). Elevations ranging from 2700 to 5600 m, with particular suitability between 3200 and 5200 m, were identified as optimal for *P. tatsienense* (Figure 4b). Precipitation during the warmest season (Bio18) between 150 and 480 mm, with 300 mm being the most suitable, was recorded as ideal for *P. tatsienense* (Figure 4c).

**TABLE 1:** The percentage contribution and permutation importance of environmental variables included in the Maxent model for predicting the distribution of *P. tatsienense* under historical, current, and future climate scenarios.

Environment variable	Describe	Percent contribution (%)	Permutation importance (%)
UVB3	Mean UV‐B of highest month	39.7	11.9
Elve	Elevation	28.7	0.2
Bio18	Precipitation of warmest quarter	17.4	16.9
Bio15	Precipitation seasonality (coefficient of variation)	6.2	7.5
UVB2	UV‐B Seasonality	5.9	56.2
Bio11	Mean Temperature of Coldest Quarter	1.6	6.7
Scb	Soil organic carbon	0.3	0.4
Bio7	Temperature annual range	0.2	0
SpH	Soil pH	0	0.2
Bio5	Precipitation seasonality	0	0

### 3.3 Suitable Habitat Distribution of *P. tatsienense* Under Potential Historical, Current, and Future Climate Scenarios

**FIGURE 5:**
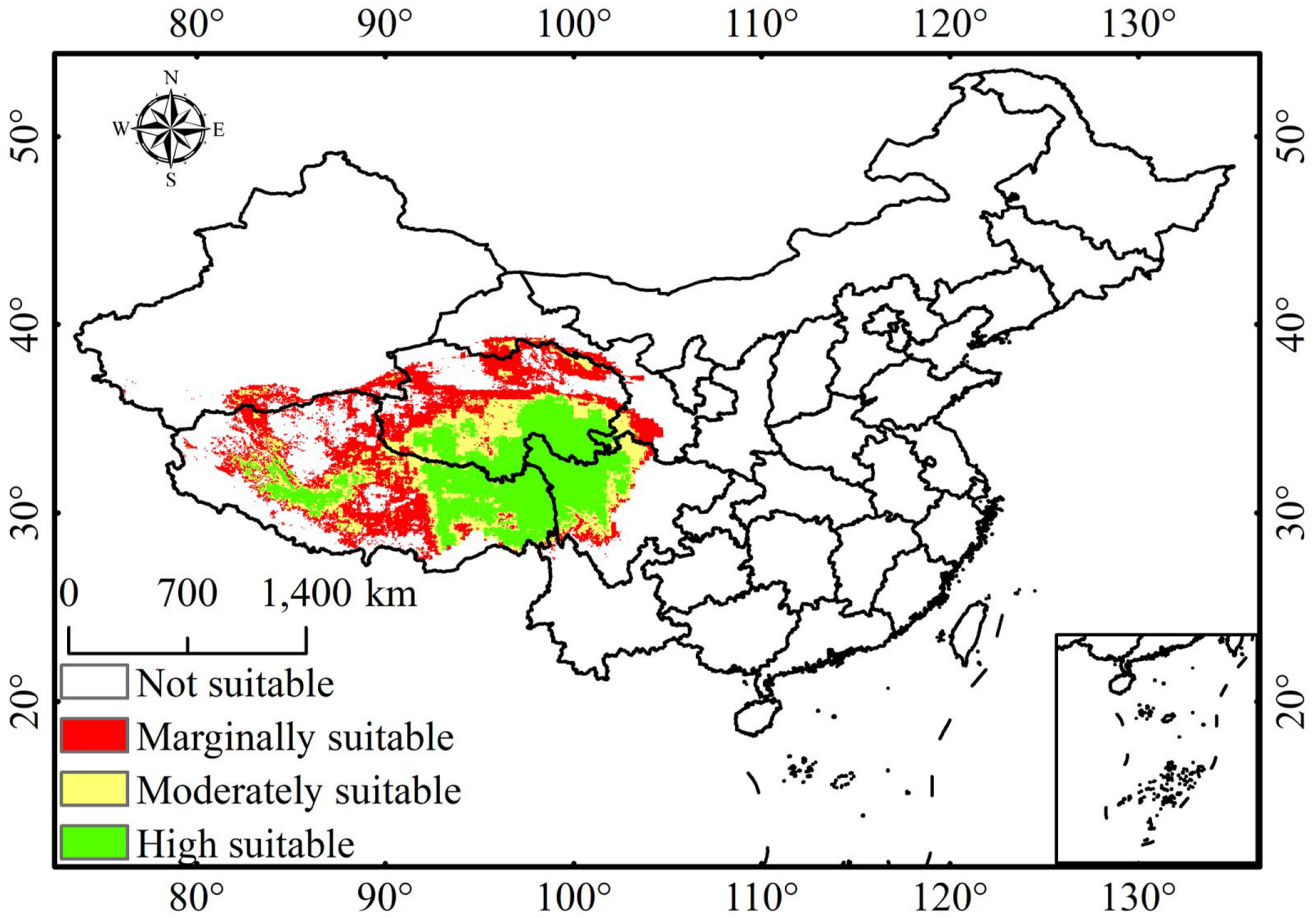
The habitat suitability of *P. tatsienense*. in China based on the Maxent model.

Figure 5 shows that the Maxent prediction closely aligns with the actual distribution of *P. tatsienense* (Figure 1). The suitability is primarily distributed in the Qinghai‐Tibet Plateau. According to the Maxent model, the highly suitable habitat for *P. tatsienense* covers 6.15 × 10^5^ km^2^ (6.40%), followed by moderately suitable habitat (4.01 × 10^5^ km^2^, 4.18%), marginally suitable habitat (4.26 × 10^5^ km^2^, 4.44%), and not suitable habitat (81.58 × 10^5^ km^2^, 84.98%) (Table 2, Figure 5). The highly suitable habitats are located at the junction of Sichuan, Tibet, and Qinghai. Moderately suitable habitats surround these areas, including northwestern Sichuan, southern Qinghai, northeastern Qinghai, and northeastern Tibet. Marginally suitable habitats are located around the moderately suitable habitats.

**TABLE 2:** Suitable distribution areas and changes in distribution area for *P. tatsienense* under historical, current, and future scenarios (×10^5^ km^2^).

Areas	Not suitable	Marginally suitable	Moderately suitable	Highly suitable
LIG	81.08	5.23	3.81	5.88
LGM	81.56	4.97	3.36	6.11
MH	79.78	6.13	3.88	6.21
Current	81.58	4.26	4.01	6.15
2050s SSP126	79.88	6.23	3.65	6.24
2050s SSP370	79.86	6.30	3.53	6.31
2050s SSP585	79.75	6.13	3.74	6.38
2070s SSP126	80.30	5.74	3.53	6.43
2070s SSP370	80.19	5.74	3.63	6.44
2070s SSP585	79.99	5.95	3.60	6.46

**FIGURE 6:**
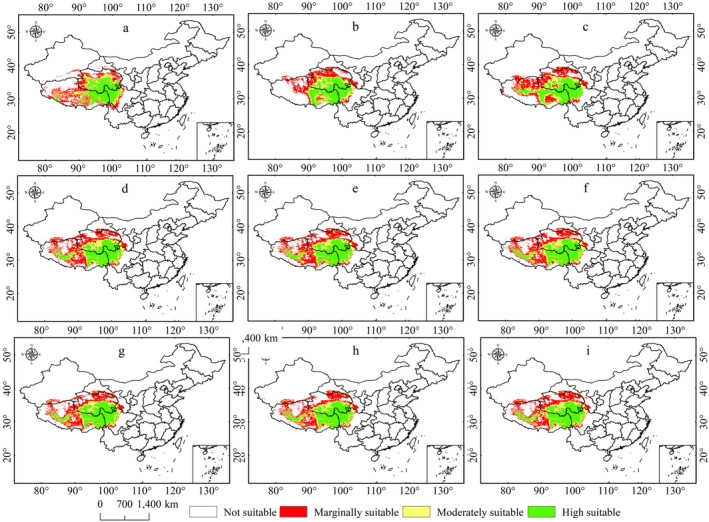
Potential distribution of *P. tatsienense* under various historical and future climate scenarios: (a) LIG; (b) LGM; (c) MH; (d) 2050s SSP126; (e) 2050s SSP370; (f) 2050s SSP585; (g) 2070s SSP126; (h) 2070s SSP370; (i) 2070s SSP585.

**FIGURE 7:**
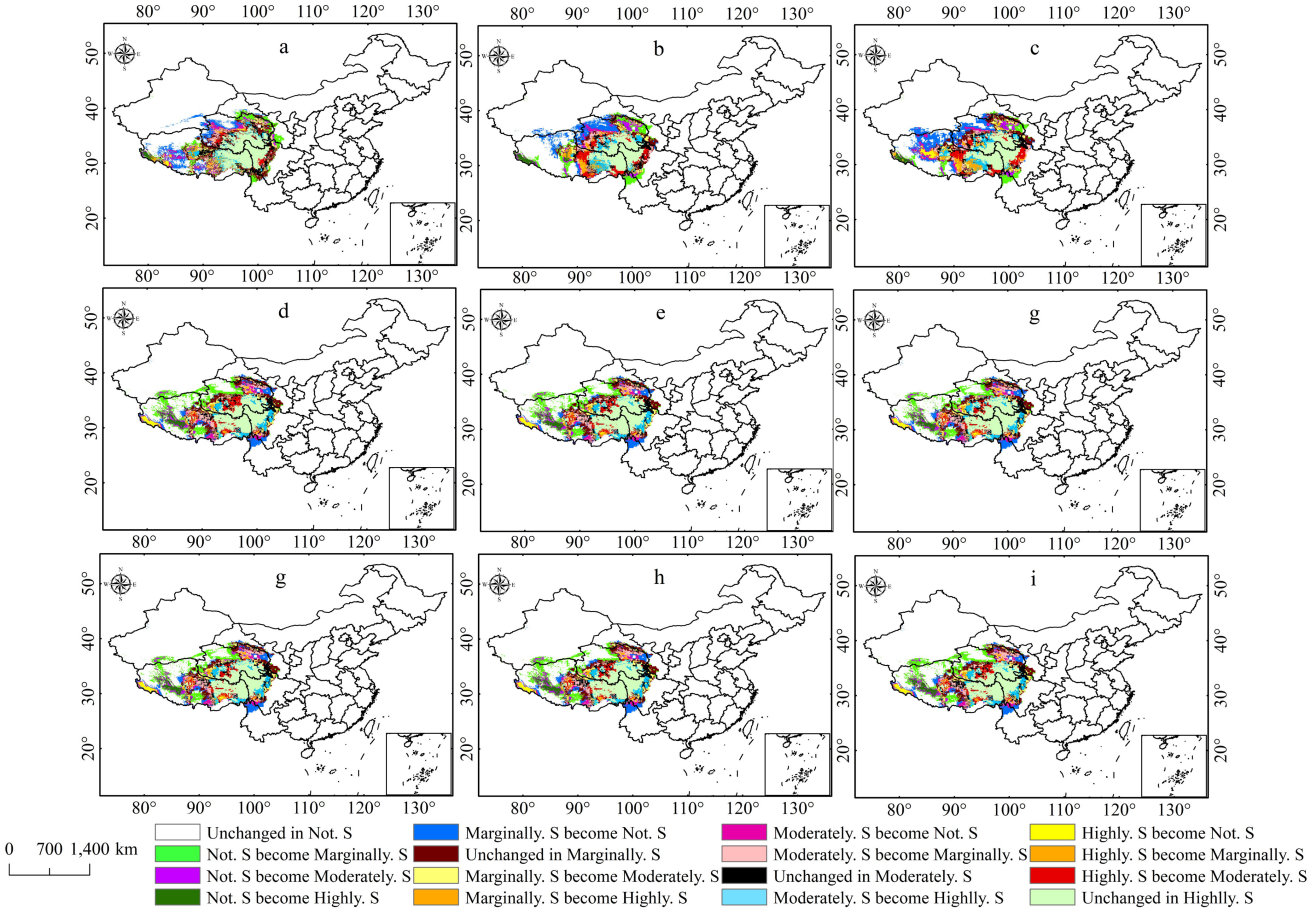
Dynamic changes in the predicted potential suitable area for *P. tatsienense* under different historical and future climate scenarios: (a) LIG; (b) LGM; (c) MH; (d) 2050s SSP126; (e) 2050s SSP370; (f) 2050s SSP585; (g) 2070s SSP126; (h) 2070s SSP370; (i) 2070s SSP585.

The study shows that the area of suitable habitat for *P. tatsienense* initially decreased from the LIG to the LGM but then increased by the MH, with the highly suitable area showing a gradual increase the presence of refugia at the junction of Sichuan, Tibet, and Qinghai (Figure 6a–c). The highly suitable habitat area peaks during the MH period at 6.21 × 10^5^ km^2^ (Table 2). Compared to the present, the suitable habitat area for *P. tatsienense* during the LIG, LGM, and MH was 0.02 × 10^5^ km^2^, 0.50 × 10^5^ km^2^, and 1.8 × 10^5^ km^2^, respectively (Table 2). The decrease in suitable habitats during the LIG relative to the current is mainly due to the transformation of marginally suitable areas in the northwestern Qinghai into unsuitable areas. During the LGM period, the reduction in suitable habitats for *P. tatsienense* was primarily due to the transformation of marginally suitable areas in northwestern Qinghai and southwest Tibet into unsuitable habitats. In addition, the greatest reduction in suitable habitat during the MH period was mainly due to the conversion of marginally suitable areas to unsuitable habitats in southwest Tibet and northwestern Qinghai (Figure 7a–c).

Future scenario predictions reveal that the distribution of suitable habitats for *P. tatsienense* will positively change, with increases in marginally and highly suitable areas from the present to the future, while moderately suitable habitats will decline. During the 2050s and 2070s, the predicted area of potentially suitable and highly roostable areas of *P. tatsienense* increases with increasing radiative forcing (Table 2; Figure 6). However, the rate of change for moderately suitable regions is slightly lower under the SSP585 scenario compared to SSP370 and SSP126 in the 2050s and 2070s. In each of the 2050s and 2070s scenarios, there was more area of marginally suitable habitat than at present (Figures 6 and 7). In the 2050s and 2070s, the largest areas of highly suitable regions are under the SSP585 scenario, with areas of 6.38 × 10^5^ km^2^ and 6.46 × 10^5^ km^2^, respectively (Table 2, Figure 6f,i), representing increases of 3.70% and 4.89% compared to current conditions. This increase is primarily due to the conversion of unsuitable regions in central Tibet, marginally and moderately suitable regions in northwestern Sichuan, into highly suitable areas (Figure 7). In the 2050s and 2070s, the total area of moderately suitable regions under all three scenarios decreased compared to the present, mainly because of transitions to highly suitable regions in northeastern Qinghai and northeastern Tibet; additionally, transitions to marginally suitable regions are observed in southwestern Tibet (Figure 7). Marginally suitable regions in all three future scenarios show an increasing trend compared to the present. This is mainly due to the transition of unsuitable habitats into marginally suitable habitats in northwestern Qinghai, northern Tibet, and southwestern Tibet. The increase in suitability in the 2050s and 2070s is primarily driven by the transition of unsuitable habitats to marginally suitable and moderately suitable habitats to highly suitable. Notably, under current climate conditions, highly suitable regions in western Tibet are becoming unsuitable habitats. In the future, high‐latitude regions may become potentially suitable habitats for *P. tatsienense* (Figure 7).

**FIGURE 8:**
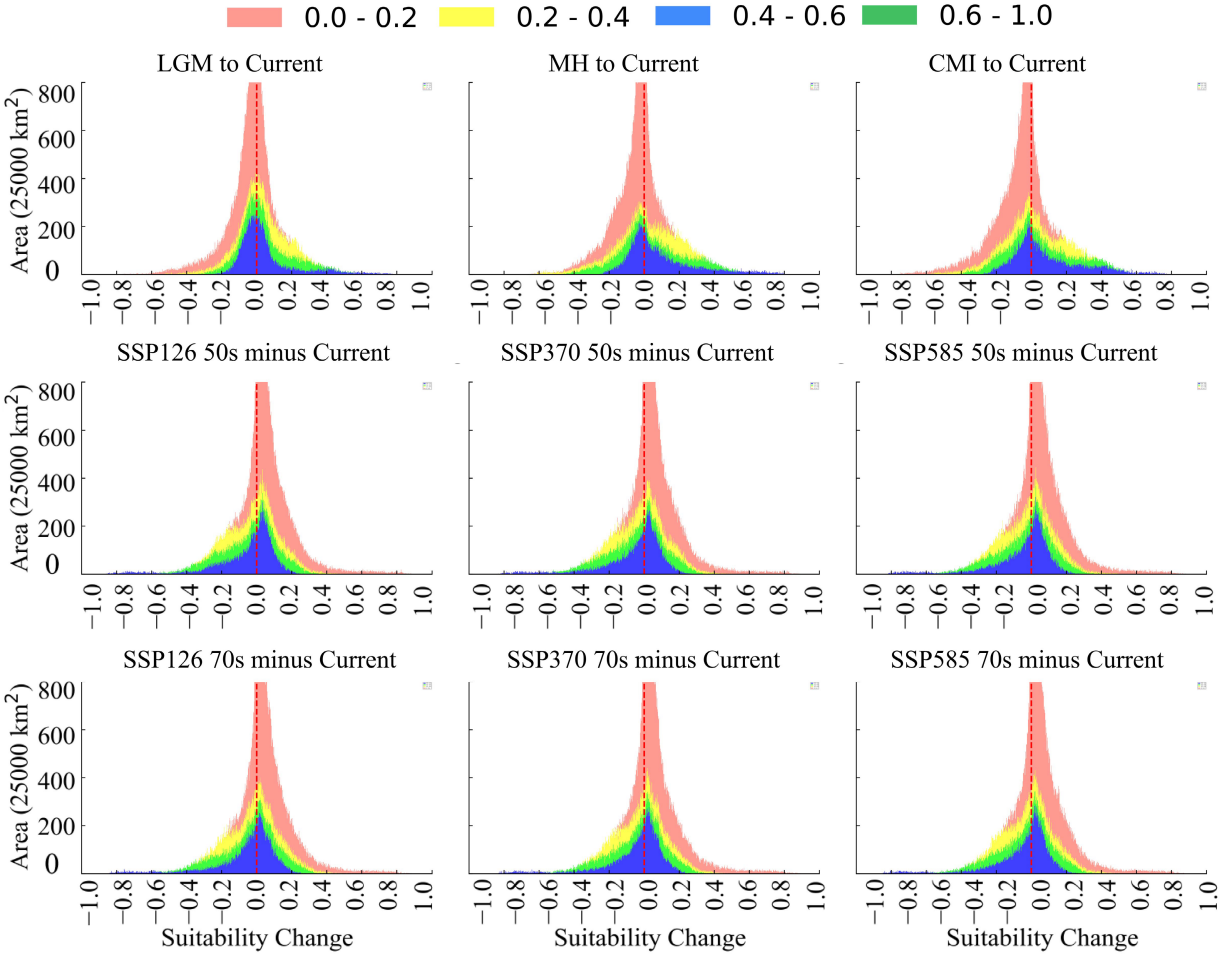
Suitability difference between current and predicted historical and future scenarios. Each color represents a category of current suitability, with the area proportional to the geographic extent of that category. Regions of suitability expansion/contraction correspond to positive/negative differences and are situated to the right/left of the vertical red line. The relationship between areas on either side of the vertical red line indicates the degree of contraction relative to expansion for each projected scenario (the x‐axis represents suitability past—suitability current and suitability current—suitability future).

This study also analyzed the expansion and contraction of suitable habitats for *P. tatsienense*, breaking them down by current suitability categories. For areas with no suitability (0–0.2), marginally suitable and highly suitable historical changes are shown on the left side of 0, while future changes are shown on the right side, indicating an increasing trend in suitable habitats from the historical to the present and into the future (Figure 8). For suitable areas (0.2–1.0), there is a noticeable change from the historical to the present. From the LIG to the present, there has been an increase of 4.36 × 10^5^ km^2^, a contraction of 9.70 × 10^5^ km^2^ from the LGM to the present, and a contraction of 13.16 × 10^5^ km^2^ from the MH to the present (Table 3). In future scenarios, under SSP126, there is an expansion of 12.58 × 10^5^ km^2^ in the 2050s but only an expansion of 6.59 × 10^5^ km^2^ in the 2070s, indicating an expected contraction of 5.99 × 10^5^ km^2^. Under SSP370, there is an expansion of 11.30 × 10^5^ km^2^ in the 2050s and 10.71 × 10^5^ km^2^ in the 2070s, with a contraction of 0.59 × 10^5^ km^2^ from 2050 to 2070. Under SSP585, the net expansion is 9.48 × 10^5^ km^2^ in the 2050s and 9.31 × 10^5^ km^2^ in the 2070s, with a net contraction of 0.17 × 10^5^ km^2^ from 2050 to 2070s (Table 3). Revealed that from 2050s to 2070s, the area of suitable habitat for *P. tatsienense* declined in each scenario.

**TABLE 3:** Predicted area of suitable habitat reduction representing contraction and expansion in different scenarios (×25 km^2^).

Scenario	Sum area C/E	Current suitability			
		0–0.2 (C/E)	0.2–0.4 (C/E)	0.4–0.6 (C/E)	0.6–1.0 (C/E)
LIG	196,991/179548	169,188/149719	6370/10677	10,024/6012	11,479/13140
LGM	207,807/169011	183,164/135952	7087/9960	7779/8257	9777/14842
MH	214,720/162076	188,353/130741	8433/8614	8024/8012	9910/14709
2050s SSP126	163,248/213570	134,555/184561	9140/7907	6764/9272	12,789/11830
2050s SSP370	165,819/210999	136,889/182227	9236/7811	6702/9334	12,992/11627
2050s SSP585	169,444/207364	140,754/178362	8990/8057	7068/8968	12,632/11987
2070s SSP126	175,233/201585	145,371/173745	9263/7784	7191/8845	13,408/11211
2070s SSP370	166,951/209777	137,811/181305	9326/7721	7126/8910	12,778/11841
2070s SSP585	169,771/207047	140,524/178592	9242/7805	7215/8821	12,790/11829

*Note:* C represents contraction, and E represents expansion.

### 3.4 Allocation and Transfer of Suitable Habitats

**FIGURE 9:**
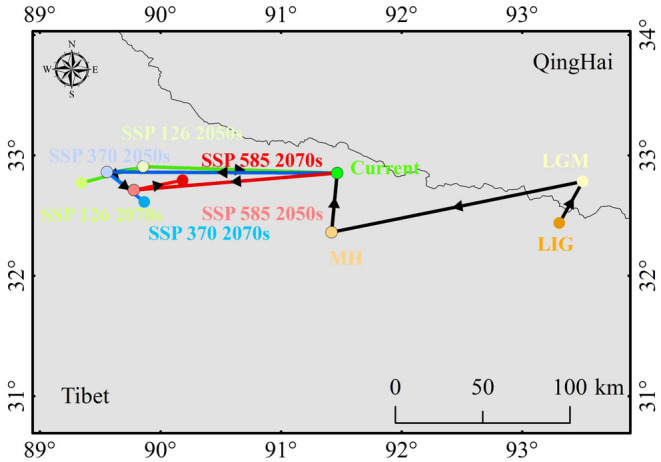
Centroids of potentially suitable habitats for *P. tatsienense* based on predictions under historical, current, and various future climate scenarios.

This study used ArcGIS to determine the centroid positions of suitable habitats for *P. tatsienense* in China (Figure 9). Currently, the centroid of these habitats is located in northern Tibet (91.47° E, 32.85° N). During the LIG, LGM, and MH, the centroids were situated in northeastern Tibet (93.31° E, 32.44° N), southern Qinghai (95.50° E, 32.78° N), and northern Tibet (91.42° E, 32.36° N), respectively. Predictions for the 2050s and 2070s suggest that the centroids of suitable habitats for *P. tatsienense* will shift further west. Under the SSP126, SSP370, and SSP585 scenarios for the 2050s, the predicted distances from current centroids are 148.33, 175.90, and 156.21 km, respectively. For the 2070s, these distances are projected to be 195.52, 150.10, and 118.22 km, respectively. During the 2050s and 2070s, the centroid of habitats under the SSP558 scenario shifted eastward compared to the SSP370 scenario.

## 4 Discussion

### 4.1 Optimized Performance of Maxent

Using 127 occurrence data points for *P. tatsienense* and 10 environmental variables, the Maxent model was optimized to predict the species' potential distribution under historical, present, and future climatic conditions. Previous studies often relied on default parameters for Maxent predictions, which led to overfitting and increased complexity, thus reducing result accuracy and sometimes generating difficult‐to‐interpret outcomes. To address this, this study optimized the Maxent model using the ENMeval package. By adjusting the regularization multiplier (RM) from 1 to 2 and changing the feature classes (FC) from LQHPT to H, the AICc value decreased from 92.95 to 0. This indicates that the optimized Maxent model effectively avoids overfitting and reliably simulates the potential geographic distribution of the species. The model's AUC value is 0.98, indicating high accuracy. The study's findings align with previous research, and the predicted potential suitable areas for *P. tatsienense* under current climatic conditions are primarily on the Qinghai‐Tibet Plateau, aligning with previous germplasm resource surveys.

### 4.2 Major Environmental Variables Affecting the Distribution of *Pyrethrum tatsienense*

According to the results of the jackknife test and contribution rates, the three main environmental factors influencing the suitability distribution of *P. tatsienense* are UVB3, Elev, and Bio18, with UVB3 being the most important. Previous studies have indicated that radiation affects vegetation growth by influencing plant stomatal conductance, photosynthesis, and the light saturation point (Zhang et al. 2018). Changes in radiation can also alter plant morphology, such as leaf thickness and the root‐to‐shoot ratio, directly impacting plant growth (Hijmans et al. 2005; Tarasov et al. 2007; Yang et al. 1994). Elevation is the second most important factor, with the suitable habitat range for *P. tatsienense* being between 2700 and 5600 m. Previous studies have reported that *P. tatsienense* grows in areas with elevations ranging from 3500 to 5200 m. This study found that *P. tatsienense* first migrates to lower latitudes and then moves to higher latitudes from the historical to the present and into the future. In historical climates, low temperatures and environmental stress at high altitudes would eliminate seedlings unable to withstand harsh conditions. Plants, under such adverse conditions, tend to limit the evolution of multiple survival mechanisms (Yang et al. 2016). Research indicates that temperatures will rise in the future, prompting some plant species to migrate to higher elevations. Seeds in high‐altitude areas have higher fat and soluble sugar content, which, combined with ample sunlight and radiation energy, may promote faster germination and early growth (Yang et al. 2016; Zhu 2002). Bio18 is another important environmental factor affecting the distribution of *P. tatsienense*, with a range of 150–480 mm, indicating that *P. tatsienense* relies on a water‐rich and warm environment. Studies have demonstrated that optimal moisture and temperature are crucial to the structure and function of vegetation and terrestrial ecosystems on the Qinghai‐Tibet Plateau (Ma and Sun 2018; Xu and Xue 2013; Xue et al. 2014; Yang et al. 2015). Insufficient moisture and poor temperature adaptability significantly reduce the photosynthetic potential of plants, directly impacting their survival (Xu and Xue 2013). Therefore, changes in these climate factors may alter the ecological suitability of *P. tatsienense*. This study found that climate changes will cause *P. tatsienense* to migrate to higher latitudes in the future.

### 4.3 Changes in the Suitability Distribution of *Pyrethrum tatsienense*

*P. tatsienense* is a unique plant and a critically endangered species in China, used in Tibetan folk medicine. Global climate change has severely impacted the distribution of *P. tatsienense*. Therefore, understanding changes in its potential distribution pattern under climate change is essential for assessing the impact on *P. tatsienense* and developing conservation strategies to maintain ecological balance. As expected, this study found that predicted habitat suitability under LIG, LGM, and MH scenarios differs from current climatic conditions, with habitat area decreasing from LIG to LGM and then increasing to MH. During the LIG, the climate was warm and humid, while the LGM had harsh conditions, and the MH climate resembled present‐day conditions (Tarasov et al. 2007; Turner et al. 2010). This indicates that compared to the LIG and LGM periods, the climate during the MH was more favorable for species expansion, and the borders of Sichuan, Tibet, and Qinghai are a refuge for *P. tatsienense*, and the climate is ideal for its growth (Lu et al. 2023). From the LIG and LGM to the MH period, the centroid of suitability shifted westward to higher altitudes. As the altitude increased, temperatures decreased linearly (Hosseini, Ghorbanpour, and Mostafavi 2024; McCutchan and Fox 1986), favoring *P. tatsienense*, which is a cold‐tolerant plant.

Future predictions of suitable areas for *P. tatsienense* under various climate scenarios reveal some variations but follow a consistent trend. Overall, there is an increase in suitable, highly suitable, and marginally suitable areas and a decrease in moderately suitable areas. The expanded areas are predominantly located in high‐latitude regions, while the decreased areas are found in western Tibet. Over time, and under future climate conditions, the centroid of suitability is expected to shift westward and to higher altitudes. Notably, across different future periods, the centroid consistently approaches the southwest junction of Tibet and the southern part of the Qinghai area, at an altitude of approximately 5000 m and with annual precipitation between 200 and 300 mm. As global temperatures continue to increase, particularly under the SSP585 scenario, where the increase is most pronounced, the centroid of *P. tatsienense* is projected to shift eastward in the 2050s and 2070s. The temperature increase from southeast to northwest suggests that excessively high temperatures will negatively impact vegetation growth. Consequently (Meng et al. 2022), while the overall suitability for *P. tatsienense* is expected to increase with rising temperatures, climate factors will continue to jointly affect its future habitat.

### 4.4 Limitations of the Study

This study selected only 10 environmental variables, which may not encompass all factors influencing the geographical distribution of *P. tatsienense*. Additional factors, such as species competition, species interactions, and human activities (e.g., overgrazing and land cover changes), could also significantly impact species distribution and introduce biases into the predictive results. Despite these limitations, this study represents the first use of an optimized Maxent model to provide reliable predictions of *P. tatsienense* distribution in China. Future research should incorporate these additional factors to improve prediction accuracy, providing more precise guidance for the conservation and management of *P. tatsienense*.

## 5 Conclusion

Accurately predicting how biological and climate variables will affect the spatial distribution of *P. tatsienense* is crucial for its conservation. Using the Maxent model, this study investigated the potential impact of future climate conditions on the habitat suitability of *P. tatsienense*. It was found that suitable areas for this species are mainly located on the Qinghai‐Tibetan Plateau, and the range of suitable habitats is projected to increase under different climate change scenarios. Key factors limiting suitable habitats include the mean UV‐B of the highest month, elevation, and warmest season precipitation. Environmental conditions in western Tibet are expected to contribute to the contraction of highly suitable habitats, while future high‐latitude regions are anticipated to become new marginally suitable habitats. These results provide valuable insights for monitoring *P*. *tatsienense* populations, estimating their distribution, and developing effective long‐term conservation strategies.

## Author Contributions

**Duo Ping Zhu:** formal analysis (equal), funding acquisition (equal), writing – original draft (equal). **Liu Yang:** funding acquisition (equal), writing – original draft (equal). **Yong‐hua Li:** data curation (equal), formal analysis (equal). **Pei Huang:** data curation (equal). **Bin Yao:** funding acquisition (equal), project administration (equal), supervision (equal), writing – original draft (equal). **Zhe Kong:** data curation (equal). **Yangzhou Xiang:** formal analysis (equal), funding acquisition (equal), writing – original draft (equal).

## Conflicts of Interest

The authors declare no conflicts of interest.

## Data Availability

The environmental variables and maps have been uploaded to an open data repository via ZENODO https://zenodo.org/records/13843418. (DOI: 10.5281/zenodo.13208470).
